# Supplementary material for: Valorisation of Wasted Immature Tomato to Innovative Fermented Functional Foods
Source: Foods. 2023 Apr 4;12(7):1532. doi: 10.3390/foods12071532 (PMC10094284; doi:10.3390/foods12071532)
Supplement: Supplementary file 1 [file foods-12-01532-s001.zip › foods-2303169-supplementary.pdf]

Supplementary Material

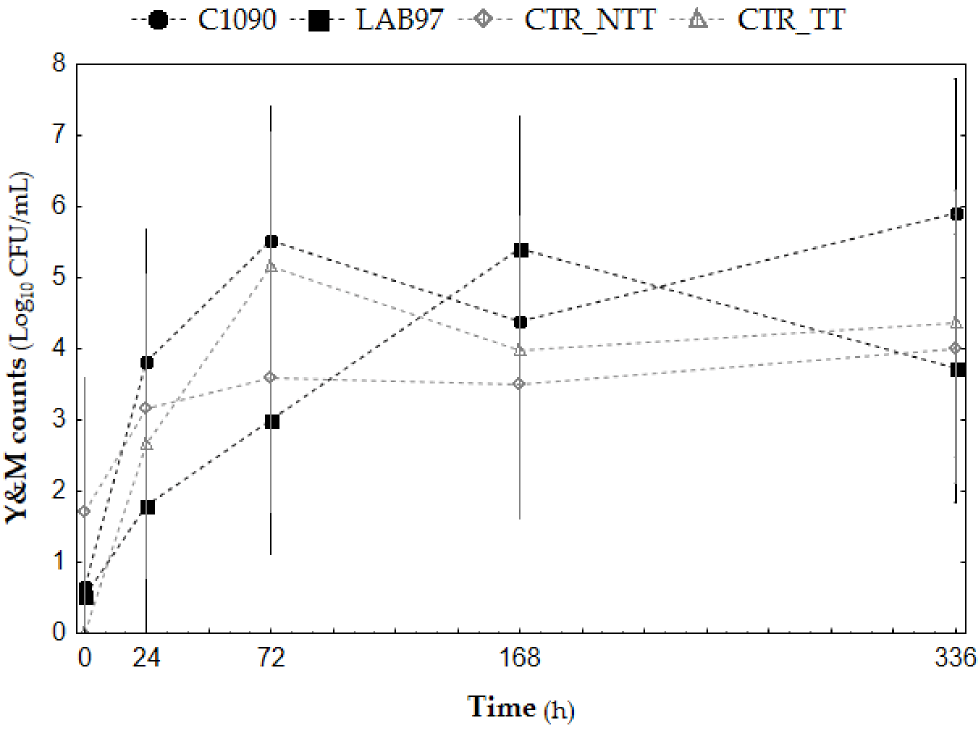

Figure S1 | Y&M counts (log_{10} CFU/mL) evolution throughout lactic acid fermentation of immature tomato pulp samples inoculated with single LAB starter cultures (LAB97 and C1090) and non-inoculated samples (CTR-NTT and CTR-TT) for 14 days. Bars represent the confidence intervals at 95%.

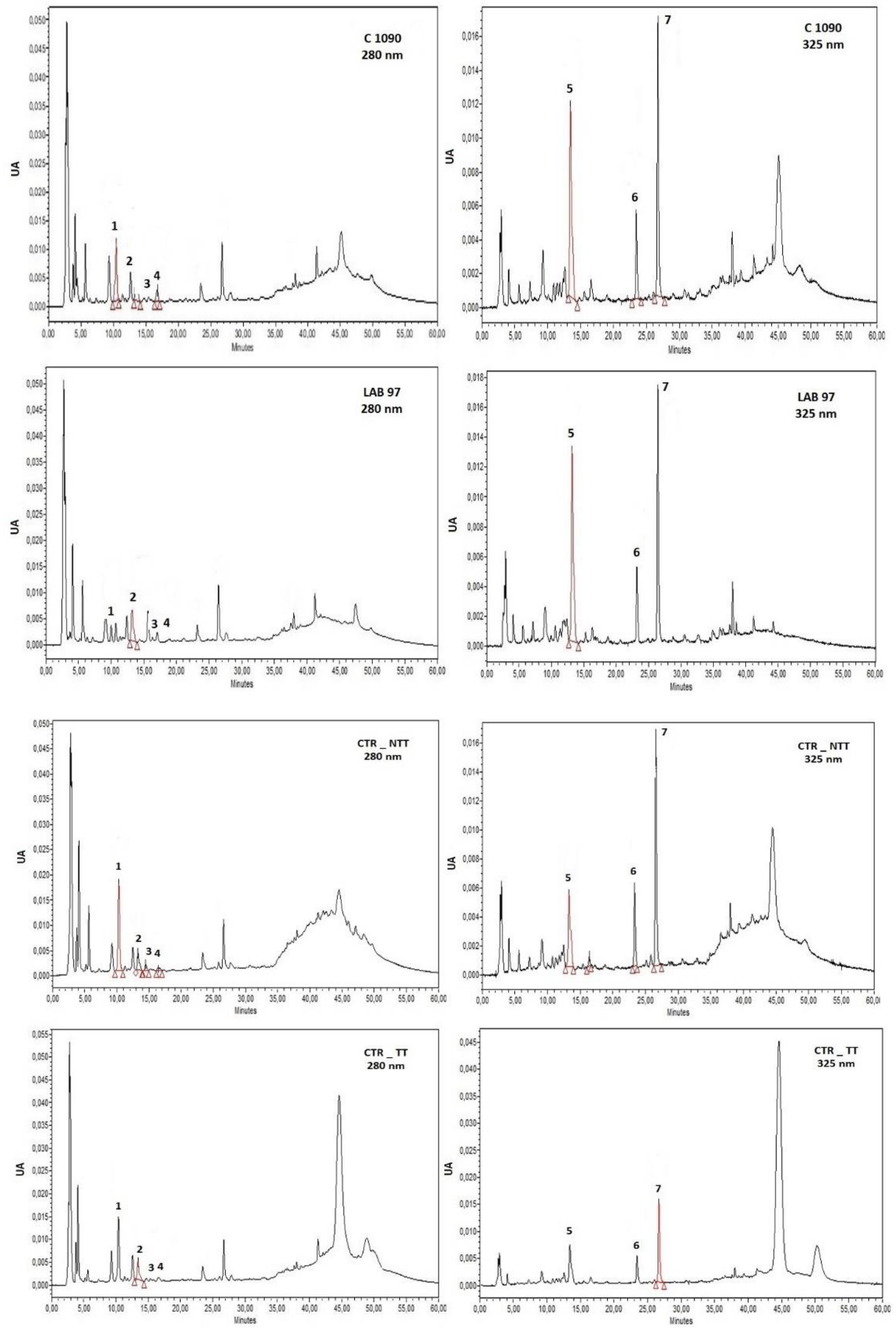

Figure S2 | HPLC-DAD chromatograms for the optimised condition at λ = 280 & 325 nm on day 7 of C1090, LAB 97, CTR _NTT and CTR _ TT samples. Peak identification: 1 - catechin; 2 - hydroxybenzoic acid; 3 - vanillic acid; 4 - syringic acid; 5 - chlorogenic acid; 6 - coumaric acid; 7 - ferulic acid.
